# C-reactive protein and mortality in 'SMuRF-less but inflamed' women and men: Evidence from the United Kingdom Biobank

**DOI:** 10.1016/j.ajpc.2026.101715

**Published:** 2026-06-25

**Authors:** Florian Kahles, Berkan Kurt, Martin Reugels, Kai M Schneider, Nikolaus Marx, Carolin V Schneider, Paul M Ridker

**Affiliations:** a Department of Internal Medicine I - Cardiology, University Hospital Aachen, RWTH Aachen University, Aachen, Germany; b Department of Internal Medicine III - Gastroenterology, University Hospital Aachen, RWTH Aachen University, Aachen, Germany; c Department of Medicine I, Gastroenterology and Hepatology, TUD Dresden University of Technology, Dresden, Germany; d Center for Regenerative Therapies Dresden (CRTD), TUD Dresden University of Technology, Dresden, Germany; e Else Kroener Fresenius Center for Digital Health, Faculty of Medicine and University Hospital Carl Gustav Carus, TUD Dresden University of Technology, Dresden, Germany; f Center for Cardiovascular Disease Prevention, Division of Preventive Medicine, Mass General Brigham, Harvard Medical School, Boston, MA, USA

**Keywords:** C-reactive protein, SMuRF, Inflammation, Primary prevention, Residual inflammatory risk, ASCVD, Sex-stratified

Residual inflammation captures a dimension of risk relevant for the development of atherosclerotic cardiovascular disease (ASCVD) and assessment of high-sensitivity C-reactive protein (hsCRP) can help to gauge this risk. As suggested by the 2025 Scientific Statement of the American College of Cardiology (ACC) on Inflammation and Cardiovascular Disease, hsCRP screening can assist in the identification of individuals prone to develop ASCVD related adverse outcomes [1]. Broadly, hsCRP levels <1, 1–3 and >3 mg/L associate with lower, intermediate, and higher residual inflammatory risk when interpreted in the context of other risk factors.

Several contemporary studies have affirmed that hsCRP, along with low-density lipoprotein cholesterol (LDL-C) and lipoprotein(a), independently predicts major adverse cardiovascular events (MACE) in the setting of primary prevention [2,3]. Recently, investigators in the United States Women’s Health Study additionally reported that individuals who lacked standard modifiable risk factors or “SMuRFs” who nonetheless had elevated levels of hsCRP were at increased lifetime risk for ASCVD events, a condition termed “SMuRF-less but Inflamed” [4]. To date, however, the generalizability of these findings beyond this female-only cohort remains uncertain.

We elected to address this issue within the population-based United Kingdom (UK) Biobank study that enrolled >500,000 adults between 2006 and 2010. Data were extracted from hospital admission records and national registries. Individuals aged 40 years or older without known ASCVD and who were free of standard modifiable risk factors (SMuRF-less) at baseline were included in this analysis. Participants were classified as SMuRF-less based on the original definition [4] if they met the following criteria: no known history of hypertension, dyslipidemia, diabetes mellitus, no current tobacco smoking, systolic blood pressure (SBP) <140 mmHg, diastolic blood pressure (DBP) <90 mmHg, total cholesterol ≤213 mg/dL, LDL-C ≤ 135 mg/dL, HbA1c <6.5% and glucose <7.0 mmol/L. Serum concentrations of hsCRP were measured using a high-sensitivity immunoturbidimetric assay. Analyses included an interaction term between hsCRP and sex to assess sex-specific effects, allowing comparability with previously published data [4]. The primary outcome was all-cause death, secondary endpoints included a composite of CV death, non-fatal myocardial infarction, non-fatal stroke (major adverse CV events, MACE) as well as CV death. Associations with hsCRP were evaluated with Kaplan-Meier analyses and complete case multivariable Cox proportional hazards models using restricted cubic splines for hsCRP, estimated glomerular filtration rate (eGFR) and interaction terms where appropriate, with hazard ratios (HR) and 95% confidence intervals (CI) reported. For sensitivity analyses a stricter SMuRF-less status was modelled as a dichotomous effect modifier by inclusion of an interaction term to avoid loss of precision. Adjusted HRs were then derived from model-based contrasts using hsCRP contrast values corresponding to the median hsCRP levels within the <1 mg/L and >3 mg/L categories, stratified by sex.

During a median follow-up of 15.3 years, all-cause death was recorded in 3323 of 67,489 SMuRF-less subjects, of which the median age was 52 [45, 60] and 59.7% were female. MACE occurred in 2000 and CV death in 411 subjects. As shown in Fig. 1, in SMuRF-less women and men, those with intermediate (hsCRP 1–3 mg/L) and high (hsCRP >3 mg/L) residual inflammatory risk had higher risk of death than those with hsCRP levels <1 mg/L (log-rank p < 0.0001). In univariable Cox regression analyses, hsCRP levels above 3 mg/L were associated with a higher risk of death in SMuRF-less women (1444 events; 3.6% event rate; HR: 2.21; 95% CI: 2.01–2.43) and men (1879 events; 6.9% event rate; HR: 2.22; 95% CI: 2.02–2.44) compared with levels <1 mg/L (p < 0.0001), with no difference between sexes (p for interaction 0.69). These results remained significant after multivariable adjustment for age, body mass index (BMI) and eGFR (p < 0.0001) for women (HR: 1.54; 95% CI: 1.39–1.71) and men (HR: 1.61; 95% CI: 1.46–1.78; p for interaction 0.14). Sensitivity analyses including lower blood pressure and LDL-C thresholds based on current US-American (SBP <130 mmHg, DBP <80 mmHg, LDL-C < 100 mg/dL) and European guidelines (SBP <120 mmHg, DBP <70 mmHg, LDL-C < 116 mg/dL) showed consistent results in women (US: HR: 1.61; 95% CI: 1.44–1.80; EU: HR: 1.63; 95% CI: 1.45–1.84) and men (US: HR: 1.69; 95% CI: 1.51–1.89; EU: HR: 1.70; 95% CI: 1.52–1.91) (all p < 0.0001).

**Fig. 1:**
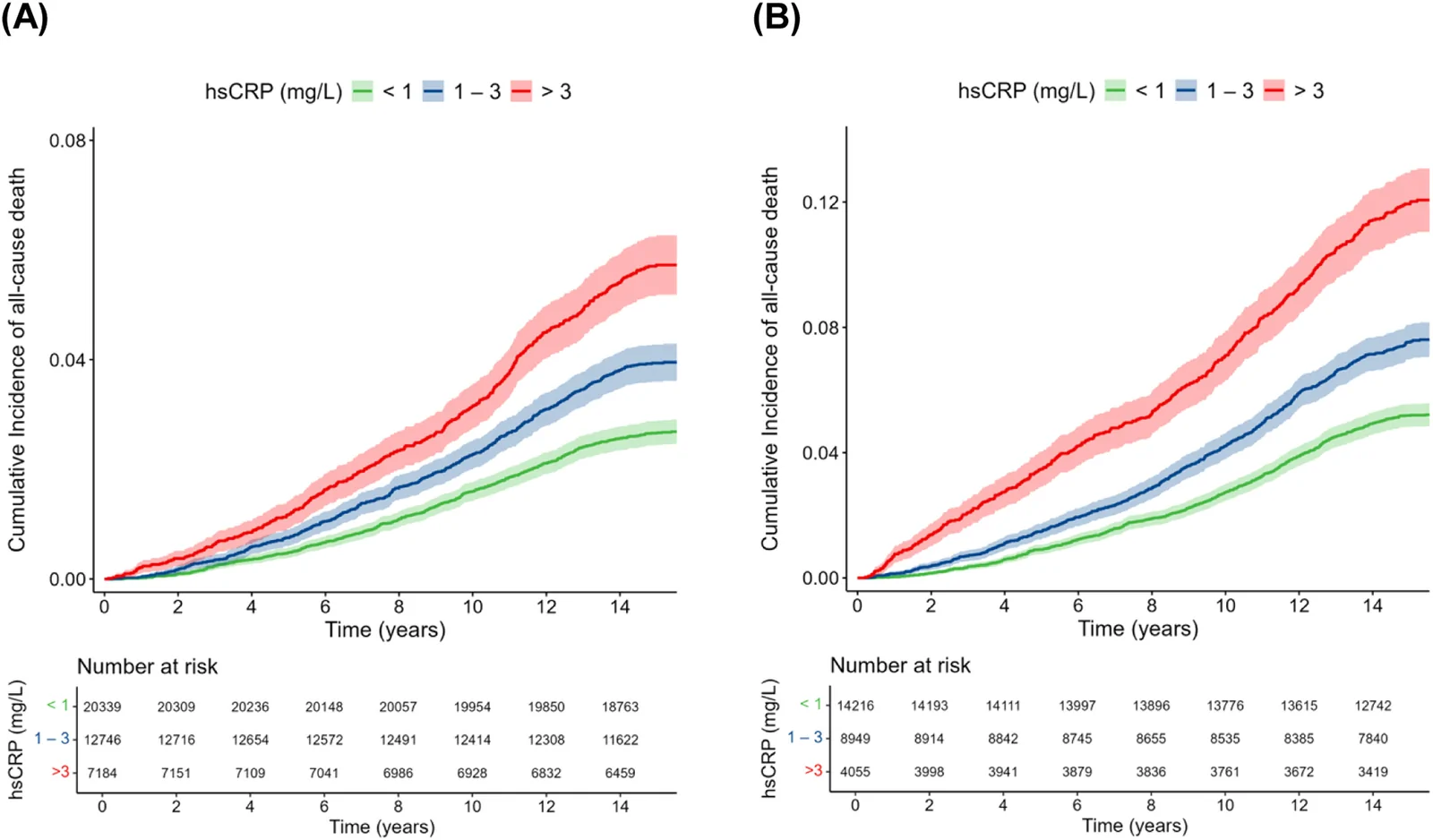
(*Central illustration*) Incidence of all-cause death in SMuRF-less subjects. Kaplan-Meier curves illustrating the cumulative incidence of all-cause death stratified by hsCRP categories of <1 mg/L vs. 1–3 mg/L vs. >3 mg/L in “SMuRF-less” women **(A)** and men **(B)**. The number at risk is displayed below the x-axis and shaded areas represent 95% confidence intervals. *Abbreviations: hsCRP: high-sensitivity C-reactive protein; SMuRF: standard modifiable cardiovascular risk factors*.

Notably, all associations were consistent for MACE and CV death in women (MACE: 745 events; 1.9% event rate; adj. HR: 1.22; 95% CI: 1.04–1.44; CV death: 136 events; 0.3% event rate; adj. HR: 1.33; 95% CI: 0.98–1.79) and men (MACE: 1255 events; 4.6% event rate; adj. HR: 1.17; 95% CI: 1.01–1.36; CV death: 275 events; 1.0% event rate; adj. HR: 1.40; 95% CI: 1.05–1.87; p for interaction: MACE: 0.47, CV death: 0.68).

The current data from UK Biobank provide confirmatory evidence that hsCRP associates with increased lifetime risk for ASCVD and all-cause mortality even among individuals lacking traditional risk factors. Importantly, the current data also extend prior findings in apparently healthy women [4] to now include apparently healthy men. For such “SMuRF-less but Inflamed” individuals, it would seem prudent for clinicians to encourage early preventive strategies including behavioral and lifestyle interventions targeting nutrition, physical activity, body weight management and smoking cessation [1,5]. Beyond such primordial prevention, recent analysis of “SMuRF-less but Inflamed” participants in the global JUPITER trial demonstrated that random allocation to rosuvastatin 20 mg daily as compared to placebo lowered cardiovascular event rates by 38% (HR 0.62; 95% CI: 0.45–0.85) [6]. Thus, for some “SMuRF-less but Inflamed” patients, physicians may also want to consider prophylactic statin therapy.

In conclusion, in a large primary prevention cohort of SMuRF-less women and men, higher hsCRP levels were associated with increased risks of long-term mortality and MACE. These findings extend prior observations beyond female-only cohorts and support broader screening of hsCRP to assist in the detection of individuals otherwise classified as low risk by traditional factors.

## Data availability

Individual-level data can be accessed through the UK Biobank upon application and approval (www.ukbiobank.ac.uk). Summary statistics generated in this study are available from the corresponding author upon reasonable request.

## Funding

F.K. was funded by the Deutsche Forschungsgemeinschaft (DFG, German Research Foundation, 520275106, Emmy Noether Research Group; SFB TRR 219; Project-ID 322900939 [M-07]), the European Research Area Network on Cardiovascular Diseases (ERA-CVD and BMBF, Grant No. JTC-2019, MyPenPath - 01KL2004), the European Foundation for the Study of Diabetes (EFSD)/Novo Nordisk Foundation (NNF2OSA0066111) and the Interdisciplinary Centre for Clinical Research within the faculty of Medicine at the 10.13039/501100007210RWTH Aachen University (IZKF project PTD 1-11). K.M.S. is supported by the Federal Ministry of and Research (BMBF) and the Ministry of Culture and Science of the German State of North Rhine-Westphalia under the Excellence strategy of the federal government and the Laender and by the CRC 1382 project B09 funded by Deutsche Forschungsgemeinschaft (DFG, German Research Foundation)—Project-ID 403224013—SFB 1382. N.M. is supported by the Deutsche Forschungsgemeinschaft (German Research Foundation; TRR 219; Project-ID 322900939 [M03, M05].

## CRediT authorship contribution statement

**Florian Kahles:** Conceptualization, Data curation, Formal analysis, Investigation, Methodology, Project administration, Resources, Supervision, Visualization, Writing – original draft, Writing – review & editing. **Berkan Kurt:** Conceptualization, Data curation, Formal analysis, Investigation, Methodology, Project administration, Visualization, Writing – original draft, Writing – review & editing. **Martin Reugels:** Conceptualization, Data curation, Formal analysis, Investigation, Methodology, Visualization, Writing – original draft, Writing – review & editing. **Kai M Schneider:** Conceptualization, Methodology, Project administration, Resources, Writing – review & editing. **Nikolaus Marx:** Conceptualization, Formal analysis, Investigation, Supervision, Writing – review & editing. **Carolin V Schneider:** Conceptualization, Formal analysis, Funding acquisition, Investigation, Project administration, Resources, Supervision, Writing – review & editing. **Paul M Ridker:** Conceptualization, Formal analysis, Investigation, Supervision, Writing – original draft, Writing – review & editing.

## Declaration of competing interest

The authors declare the following financial interests/personal relationships which may be considered as potential competing interests.

F.K. has served as a speaker for Novo Nordisk, Boehringer Ingelheim, Bayer, Amgen, Apontis Pharma, Eli Lilly, AstraZeneca, C.T.I., Cogitando, Synlab, CSL Vifor, DGK-Akademie, SphingoTec, 4TEEN4 Pharmaceuticals and diaplan GmbH, consulted Novo Nordisk, Eli Lilly, Bayer, Apontis Pharma, Pricewaterhouse Coopers/ Strategy&, received travel support from Amgen, Novo Nordisk, Boehringer Ingelheim, Bayer, SphingoTec, 4TEEN4 Pharmaceuticals, diaplan GmbH, C.T.I., Cogitando, and Eli Lilly and has received research-related in-kind services from SphingoTec and 4TEEN4 Pharmaceuticals. B.K. has served as a speaker for Novo Nordisk, Lilly, Apontis Pharma, SphingoTec, 4TEEN4 Pharmaceuticals, DGK-Akademie, and the Professional Association of German Internists (BDI e.V.), has consulted for Novo Nordisk and Roche Diagnostics, has received travel support from Novo Nordisk, Lilly, Boehringer Ingelheim, AstraZeneca, Apontis Pharma, Daiichi Sankyo, Edwards Lifesciences, Medtronic, 4TEEN4 Pharmaceuticals, the German Society of Lipidology, DGK-Akademie and the German Cardiac Society and has received research-related in-kind services from SphingoTec and 4TEEN4 Pharmaceuticals. N.M. has given lectures for Amgen, Bayer, Boehringer Ingelheim, Sanofi-Aventis, MSD, BMS, AstraZeneca, Lilly, Novo Nordisk; has received unrestricted research grants from Boehringer Ingelheim, and has served as an advisor for Bayer, Boehringer Ingelheim, Sanofi-Aventis, MSD, BMS, AstraZeneca, Novo Nordisk. In addition, served in trial leadership for Boehringer Ingelheim and Novo Nordisk. N.M. declines all personal compensation from pharma or device companies. P.M.R. has received institutional research grant support from Kowa, Novartis, Amarin, Pfizer, Esperion, Novo Nordisk, and the NHLBI; during the past 3 years has served as a consultant to Novartis, Agepha, Arrowhead, AstraZeneca, CSL Behring, Civi Biopharma, Merck, SOCAR, Novo Nordisk, Eli Lilly, New Amsterdam, Boehringer Ingelheim, Cytokinetics, Nodthera, Tourmaline Bio, and Cardio Therapeutics; has minority shareholder equity positions in Uppton, Bitteroot Bio, and Angiowave; and receives compensation for service on the Peter Munk Advisory Board (University of Toronto), the Leducq Foundation, Paris, France.
